# Direct comparison and reproducibility of two segmentation methods for multicompartment dosimetry: round robin study on radioembolization treatment planning in hepatocellular carcinoma

**DOI:** 10.1007/s00259-023-06416-9

**Published:** 2023-09-12

**Authors:** Marnix Lam, Etienne Garin, Xavier Palard-Novello, Armeen Mahvash, Cheenu Kappadath, Paul Haste, Mark Tann, Ken Herrmann, Francesco Barbato, Brian Geller, Niklaus Schaefer, Alban Denys, Matthew Dreher, Kirk D. Fowers, Vanessa Gates, Riad Salem

**Affiliations:** 1 Department of Radiology and Nuclear Medicine, University Medical Center Utrecht, Utrecht, The Netherlands; 2 Nuclear Medicine Department, Eugene Marquis Center, Rennes, France; 3 Department of Interventional Radiology, University of Texas MD Anderson Cancer Center, Houston, TX USA; 4 Department of Clinical Radiology and Imaging Sciences, Indiana University School of Medicine, Indianapolis, IN USA; 5 Department of Nuclear Medicine, University of Duisburg-Essen, and German Cancer Consortium (DKTK)-University Hospital Essen, Essen, Germany; 6 Department of Radiology, University of Florida, Gainesville, FL USA; 7 Department of Nuclear Medicine and Molecular Imaging, Lausanne University Hospital CHUV, University of Lausanne, Lausanne, Switzerland; 8 Department of Radiology and Interventional Radiology, Lausanne University Hospital CHUV, University of Lausanne, Lausanne, Switzerland; 9 Boston Scientific Corporation, Marlborough, MA USA; 10 Department of Radiology, Northwestern Feinberg School of Medicine, Chicago, IL USA

**Keywords:** Radioembolization, Yttrium-90, Dosimetry, Hepatocellular carcinoma

## Abstract

**Purpose:**

Investigate reproducibility of two segmentation methods for multicompartment dosimetry, including normal tissue absorbed dose (NTAD) and tumour absorbed dose (TAD), in hepatocellular carcinoma patients treated with yttrium-90 (^90^Y) glass microspheres.

**Methods:**

TARGET was a retrospective investigation in 209 patients with < 10 tumours per lobe and at least one tumour ≥ 3 cm ± portal vein thrombosis. Dosimetry was compared using two distinct segmentation methods: anatomic (CT/MRI-based) and count threshold-based on pre-procedural ^99m^Tc-MAA SPECT. In a round robin substudy in 20 patients with ≤ 5 unilobar tumours, the inter-observer reproducibility of eight reviewers was evaluated by computing reproducibility coefficient (RDC) of volume and absorbed dose for whole liver, whole liver normal tissue, perfused normal tissue, perfused liver, total perfused tumour, and target lesion. Intra-observer reproducibility was based on second assessments in 10 patients ≥ 2 weeks later.

**Results:**

^99m^Tc-MAA segmentation calculated higher absorbed doses compared to anatomic segmentation (*n* = 209), 43.9% higher for TAD (95% limits of agreement [LoA]: − 49.0%, 306.2%) and 21.3% for NTAD (95% LoA: − 67.6%, 354.0%). For the round robin substudy (*n* = 20), inter-observer reproducibility was better for anatomic (RDC range: 1.17 to 3.53) than ^99m^Tc-MAA SPECT segmentation (1.29 to 7.00) and similar between anatomic imaging modalities (CT: 1.09 to 3.56; MRI: 1.24 to 3.50). Inter-observer reproducibility was better for larger volumes. Perfused normal tissue volume RDC was 1.95 by anatomic and 3.19 by ^99m^Tc-MAA SPECT, with corresponding absorbed dose RDC 1.46 and 1.75. Total perfused tumour volume RDC was higher, 2.92 for anatomic and 7.0 by ^99m^Tc-MAA SPECT with corresponding absorbed dose RDC of 1.84 and 2.78. Intra-observer variability was lower for perfused NTAD (range: 14.3 to 19.7 Gy) than total perfused TAD (range: 42.8 to 121.4 Gy).

**Conclusion:**

Anatomic segmentation-based dosimetry, versus ^99m^Tc-MAA segmentation, results in lower absorbed doses with superior reproducibility. Higher volume compartments, such as normal tissue versus tumour, exhibit improved reproducibility.

**Trial registration:** NCT03295006.

**Supplementary Information:**

The online version contains supplementary material available at 10.1007/s00259-023-06416-9.

## Introduction

Transarterial radioembolization (TARE) using yttrium-90 (^90^Y) glass microspheres (TheraSphere™, Boston Scientific Corporation, Marlborough, MA, USA) is a well-established locoregional treatment option for patients with hepatocellular carcinoma (HCC) [1]. ^90^Y glass microspheres are 15 to 35 µm in size. They are delivered into the liver through a microcatheter placed into the hepatic artery that supplies blood to the tumour. Emitted beta radiation exerts a local radiotherapeutic effect, which is delivered over approximately two weeks post-treatment, while the inert glass microspheres remain permanently implanted.

Studies demonstrated that TARE improves patient outcomes, including overall survival (OS), where dosing is personalized with a net increase in the tumour absorbed dose (TAD) [2–9]. Personalization can be achieved by more selective infusion and/or through multicompartment dosimetry, also known as partition modeling. Ensuring minimal normal tissue absorbed dose (NTAD) and adequate hepatic reserve are key safety considerations in treating HCC patients [10]. Personalized treatment can be implemented by analyzing intra-arterially injected technetium-99m (^99m^Tc) macroaggregated albumin (MAA) distribution on pre-procedural SPECT/CT, calculating anticipated ^90^Y TAD and NTAD on an individual patient basis [1, 11].

In contrast to a single-compartment dosimetry approach based on the mean absorbed dose to the target or perfused volume (without taking TAD and NTAD into account), the TheraSphere™ Advanced Dosimetry Retrospective Global Study Evaluation in Hepatocellular Carcinoma Treatment (TARGET) study retrospectively assessed the value of an alternative multicompartment dosimetry methodology to calculate TAD and NTAD, both based on the medical internal radiation dose (MIRD) schema [4, 10, 12–17]. This method requires the definition of volumes of interest (e.g., tumour volume, normal tissue volume) and quantification of ^99m^Tc-MAA activity within these volumes to finally arrive at anticipated ^90^Y mean absorbed doses (e.g., TAD and NTAD). Definition of the volume of interest or segmentation is usually performed on baseline contrast-enhanced CT or MRI but can also be done using functional imaging by delineation/thresholding of the counts within that volume on ^99m^Tc-MAA SPECT/CT [9, 18]. The DOSISPHERE-01 study, as well as other studies, utilized such a ^99m^Tc-MAA SPECT count threshold-based segmentation method to guide volumes of interest, which assumes that ^99m^Tc-MAA preferentially accumulates in the tumour [9, 17]. Other studies used anatomic images (i.e., CT/MRI) to define volumes of interest [4, 11, 16, 18–20]. The TARGET study evaluated both anatomic and ^99m^Tc-MAA SPECT segmentation methods to compare the real-world utility for ^90^Y treatment planning [7, 9].

Besides the accurate definition of volumes of interest, pre-treatment ^99m^Tc-MAA-based planning also requires sufficient predictive power for final ^90^Y distribution. Currently, there are no validated methods to consistently estimate TAD and NTAD using ^99m^Tc-MAA as a viable surrogate; however, numerous publications have investigated and confirmed the utility of ^99m^Tc-MAA [4, 9–11, 15–17, 19, 21]. The European Association of Nuclear Medicine dosimetry committee 2021 clinical guidelines recommend the calculation of absorbed doses both pre-treatment using ^99m^Tc-MAA and post-treatment using ^90^Y imaging, with distinct evaluation between target tumours and normal tissue for treatment optimization [18]. However, no guidance is provided with regard to the volume of interest definition or segmentation.

Real-world utility of any dosimetry method depends on the ability of clinicians to reliably and accurately determine the anticipated and real absorbed dose to achieve personalization of treatment. Here, we present a patient-by-patient comparison of the two segmentation methods for total perfused TAD and NTAD, as well as inter- and intra-observer reproducibility results of the TARGET study. The TARGET study was an international, multi-center, retrospective, single-arm study of patients from 13 centers located across eight countries who were treated using ^90^Y glass microspheres for HCC. The study consisted of three parts: (1) collect clinical data to generate predictive models for NTAD and TAD association with clinical outcomes, (2) evaluate the inter-site variability of imaging systems using phantom studies, and (3) evaluate dosimetry software/methodology reproducibility among reviewers and comparing different segmentation methods, which is the focus of this manuscript, also known as the round robin substudy [7].

While several single-center studies have evaluated reproducibility [4, 15–17, 19–22], this is the largest real-world global evaluation of personalized dosimetry reproducibility. The aim was to understand the differences in dosimetry results between both methods used, as well as the reliability in terms of reproducibility across physicians and sites. This will help to compare published study results that used either one of the two presented methods.

## Material and methods

### Study design and inclusion/exclusion criteria

In the TARGET study, patients with < 10 well-defined HCC tumours per lobe with at least one tumour ≥ 3 cm ± portal vein thrombosis (PVT) were included [7]. Protocols were approved by each site’s respective Institutional Review Boards (IRBs) and/or Independent Ethics Committees (IECs). Imaging was based on institutional practice, but required, at a minimum, diagnostic contrast-enhanced imaging (CT or MRI) and a two-headed SPECT camera system using ^99m^Tc-MAA SPECT/CT. Each patient had two different methods of segmentation performed: anatomic and ^99m^Tc-MAA segmentation. The two segmentation methods were compared for total perfused TAD and NTAD for the full TARGET study population (*n* = 209). The first 20 eligible patients, with ≤ 5 unilobar tumours, submitted by participating centers, were enrolled in the round robin substudy, which evaluated inter-observer (*n* = 20) and intra-observer (*n* = 10) reproducibility using Simplicit^90^Y™ dosimetry software (Version 1.1, Mirada Medical Ltd.) to calculate multicompartment dosimetry, including the NTAD and TAD, in HCC patients by eight reviewers (with each reviewer at a different clinical site). Individuals had no or limited experience with the dosimetry software prior to initiation of the TARGET round robin substudy.

Endpoints were assessed based on both the anatomic (MRI- or CT-based assessment) and the ^99m^Tc-MAA SPECT segmentation methods. In both segmentation methods, diagnostic imaging was registered to the ^99m^Tc-MAA SPECT with each of the reviewers as the arbiter of the registration quality. The intended ^90^Y absorbed dose to the perfused liver volume was set to 120 Gy for both ^99m^Tc-MAA SPECT and anatomic segmentation methods.

For ^99m^Tc-MAA SPECT segmentation, the whole liver volume was delineated using anatomic imaging, and the perfused volume and tumour volume were delineated based on a reviewer-dependent count-based threshold to best delineate the volumes of interest. The ^99m^Tc-MAA SPECT segmentation technique used in this study involved using the “% threshold” tool within Simplicit^90^Y, which was applied within a user-defined box around the desired volume as visualized on ^99m^Tc-MAA SPECT/CT. The anatomic segmentation method relied solely on anatomic segmentation on MRI or CT of the different volumes of interest.

Inter-observer reproducibility was evaluated based on each patient (*n* = 20) being assessed by eight reviewers. Intra-observer reproducibility was assessed using a subset of the first 10 patients included in the assessment of inter-observer variability, where assessment and re-assessment were performed at least two weeks apart.

### Data collected

The endpoints of interest included total administered activity (GBq), absorbed dose and volumes for whole liver, perfused liver, total perfused tumours, perfused normal tissue, whole liver normal tissue, and target tumour (i.e., single largest lesion).

### Statistics

A total of 209 patients were included in the TARGET study. A Bland–Altman analysis on log-transformed data evaluated the agreement between total perfused TAD and NTAD by segmentation method, anatomic versus ^99m^Tc-MAA, and was performed for the full clinical study population.

The assessment of inter-observer reproducibility required eight reviewers, each providing data on the same set of 20 patients. For each dosimetric endpoint, the reproducibility coefficient (RDC) was computed using the random effects model described by Raunig et al. [23]. Assuming the data has a normal distribution, the RDC provides a measure of the maximum difference of the dosimetric endpoint from two different reviewers in 95% of cases, and the associated 95% confidence interval (CI) shows the precision of the RDC value. The sample size of eight reviewers and 20 patients was determined using a simulation based on actual data on absorbed dose to the normal liver tissue previously published from Indiana University [22]. The simulation showed that this sample size would give a 95% CI for the RDC with a width < 2.2 Gy in 80% of the simulations, which was considered to provide sufficient precision for the RDC.

A pre-planned assessment indicated non-normally distributed data, and therefore, a log transformation was applied. As a consequence, the RDCs reported here provide a measure of the maximum ratio of the dosimetric endpoint from two different reviewers in 95% of cases, rather than the maximum difference. Repeat assessments performed by the same reviewers in order to measure intra-observer reproducibility were not included in the computations to measure inter-observer reproducibility. To facilitate a comparison of reproducibility over the dosimetric endpoints, grades of reproducibility were defined, post-hoc, based on the upper value of the 95% CI for the RDC (Table 3).

The following summary statistics were computed separately for each of the 20 patients for each dosimetric endpoint:

- Coefficient of variation (CV %) showed the variability across all eight reviewers.
- The mean absolute percentage error (MAPE) is computed as follows:

MAPE = $$\frac{{\sum }_{i=1}^{8}{e}_{is}}{8}$$

where *e*_*is*_ = 100 × is $\frac{\left|{y}_{is-}{\overline{y} }_{l}\right|}{{\overline{y} }_{l}}$ the absolute percentage error, and ${\overline{y} }_{l}=\frac{{\sum }_{s=1}^{8}{Y}_{is}}{8}$ is the mean over the eight reviewers and ${y}_{is}$ is the value of the dosimetric endpoint for the *i*th patient assessed by the *s*th reviewer.

Outliers were identified separately for the first and second assessments for each patient as values less than *Q*_*L*_* − 3*(*Q*_*U*_* – Q*_*L*_) or values greater than *Q*_*U*_ + *3*(*Q*_*U*_* – Q*_*L*_), where *Q*_*L*_ and *Q*_*U*_ are the lower and upper quartiles of the values over the eight reviewers, respectively. Outliers were not excluded from the analyses but were assessed to identify any patients or reviewers who had a greater frequency of outliers.

The sample size of 10 patients for the assessment of intra-observer reproducibility was not based on a sample size calculation because this was considered an exploratory analysis. Intra-observer variability (IOV) is a measurement of the variation between readings of the same patient by the same reviewer and was computed as described in the Supplementary information.

Statistical analyses were conducted using SAS® Release 9.4 (SAS Institute Inc., Cary, North Carolina, USA).

## Results

**Fig. 1:**
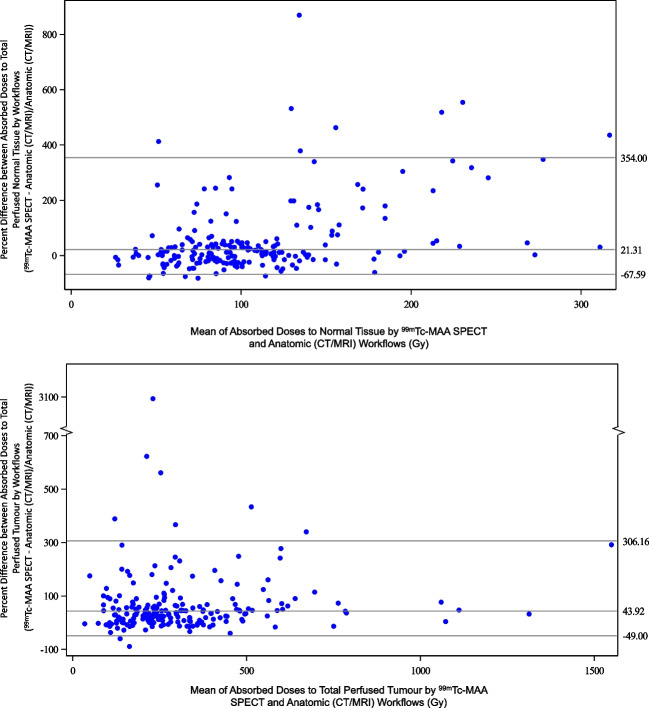
Percentage difference of NTAD (top) and total perfused TAD (bottom) by segmentation method, anatomic or ^99m^Tc-MAA segmentation. The center horizontal line shows the bias, and the horizontal lines above and below the center line show the 95% limits of agreement, as computed from a Bland–Altman analysis of log-transformed data

Included patients were treated with ^90^Y glass microspheres between 1^st^ January 2010 and 31^st^ December 2017. A total of 209 met the inclusion criteria. Detailed baseline patient characteristics were described previously [7]. In short, patients had a median age of 66 years (range 27–87 years) and were classified as BCLC A (12.9%, *n* = 27), B (32.5%, *n* = 68), or C (54.5%, *n* = 114). The majority of patients had a single tumour (69.4%, *n* = 145), unilobar (70.8%, *n* = 148) disease, and target lesion in the right lobe (85.6%, *n* = 179) ≥ 5 cm (80.4%, *n* = 168). The Bland–Altman analysis noted on average that ^99m^Tc-MAA segmentation gave 43.9% higher TAD (95% limits of agreement [LoA]: − 49.0%, 306.2%) and 21.3% higher NTAD (95% LoA: − 67.6%, 354.0%) versus anatomic segmentation (Fig. 1). Table 1 provides the RDC values for 20 patients across eight reviewers (*n* = 160) based on the two dosimetry segmentation methods for each patient. Part of the target lesion data (11/160) was excluded from analysis due to incorrect identification of the target lesion, eight patients by one reviewer, and one patient each for three additional reviewers.

**Table 1:** Inter-observer reproducibility coefficient (RDC) values

Parameter	Segmentation method			
	^99m^Tc-MAA SPECT	Anatomic CT	Anatomic MRI	Anatomic CT/MRI
Administered activity (GBq)				
*n*	160	96	64	160
RDC [95% CI]	2.10 [1.86, 3.13]	1.29 [1.25, 1.35]	1.29 [1.21, 1.60]	1.29 [1.25, 1.48]
Whole liver**,** volume (cm^3^)				
*n*	160	96	64	160
RDC [95% CI]	1.29 [1.22, 1.52]	1.09 [1.08, 1.20]	1.25 [1.18, 1.51]	1.17 [1.14, 1.27]
Absorbed dose (Gy)				
*n*	160	96	64	160
RDC [95% CI]	1.97 [1.76, 2.87]	1.27 [1.22, INF]	1.24 [1.18, 1.48]	1.25 [1.22, 1.41]
Whole liver normal tissue, volume (cm^3^)				
*n*	160	96	64	160
RDC [95% CI]	1.37 [1.31, 1.61]	1.34 [1.27, 1.63]	1.40 [1.31, 2.06]	1.36 [1.30, 1.59]
Absorbed dose (Gy)				
*n*	160	96	64	160
RDC [95% CI]	3.62 [2.98, 6.95]	1.83 [1.64, 2.79]	1.89 [1.66, 4.36]	1.85 [1.69, 2.52]
Perfused normal tissue, volume (cm^3^)				
*n*	160	96	64	160
RDC [95% CI]	3.19 [2.71, 5.66]	1.92 [1.71, 3.02]	2.00 [1.71, 3.63]	1.95 [1.75, 2.77]
Absorbed dose (Gy)				
*n*	160	96	64	160
RDC	1.75 [1.62, 2.31]	1.49 [1.40, 8.23]	1.41 [1.33, 1.52]	1.46 [1.39, 2.25]
Perfused liver,^a^ volume (cm^3^)				
*n*	160	96	64	160
RDC [95% CI]	2.12 [1.88, 3.17]	1.29 [1.25, 1.35]	1.29 [1.21, 1.60]	1.29 [1.25, 1.48]
Total perfused tumours, volume (cm^3^)				
*n*	160	96	64	160
RDC [95% CI]	7.00 [5.05, 20.25]	2.53 [2.09, 4.84]	3.50 [2.81, NC]	2.92 [2.48, 5.02]
Absorbed dose (Gy)				
*n*	160	96	64	160
RDC [95% CI]	2.78 [2.29, 5.24]	1.65 [1.49, 2.31]	2.11 [1.80, 4.32]	1.84 [1.66, 2.56]
Target lesion,^b^ volume (cm^3^)				
*n*	149	90	59	149
RDC [95% CI]	6.90 [4.95, 20.48]	3.56 [2.95, NC]	3.49 [2.85, 4.72]	3.53 [3.02, 64.08]
Absorbed dose (Gy)				
*n*	149	90	59	149
RDC [95% CI]	2.84 [2.33, 5.41]	1.69 [1.55, 3.09]	2.61 [2.15, 21.89]	2.07 [1.87, 3.10]

^a^Absorbed dose to the perfused liver was set to 120 Gy for all patients

^b^Part of the target lesion data (11/160) was excluded from analysis due to incorrect identification of the target lesion, eight patient’s data by one reviewer, and one patient each for three additional reviewers

*CI*, confidence interval; *INF*, infinite; *n*, number of observations used in analysis; *NC*, not calculable; *RDC*, reproducibility coefficient; ^*99m*^*Tc-MAA*, technetium-99m macroaggregated albumin; *SPECT*, single-photon emission computed tomography

**Fig. 2:**
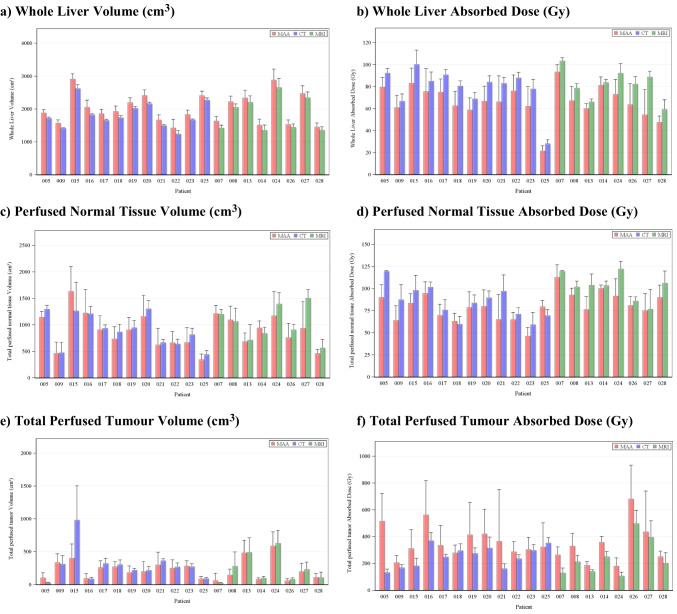
Bar charts of mean and standard deviation (as shown by error bars) over reviewers in patients with hepatocellular carcinoma treated with yttrium-90 (^90^Y) glass microspheres

**Fig. 3:**
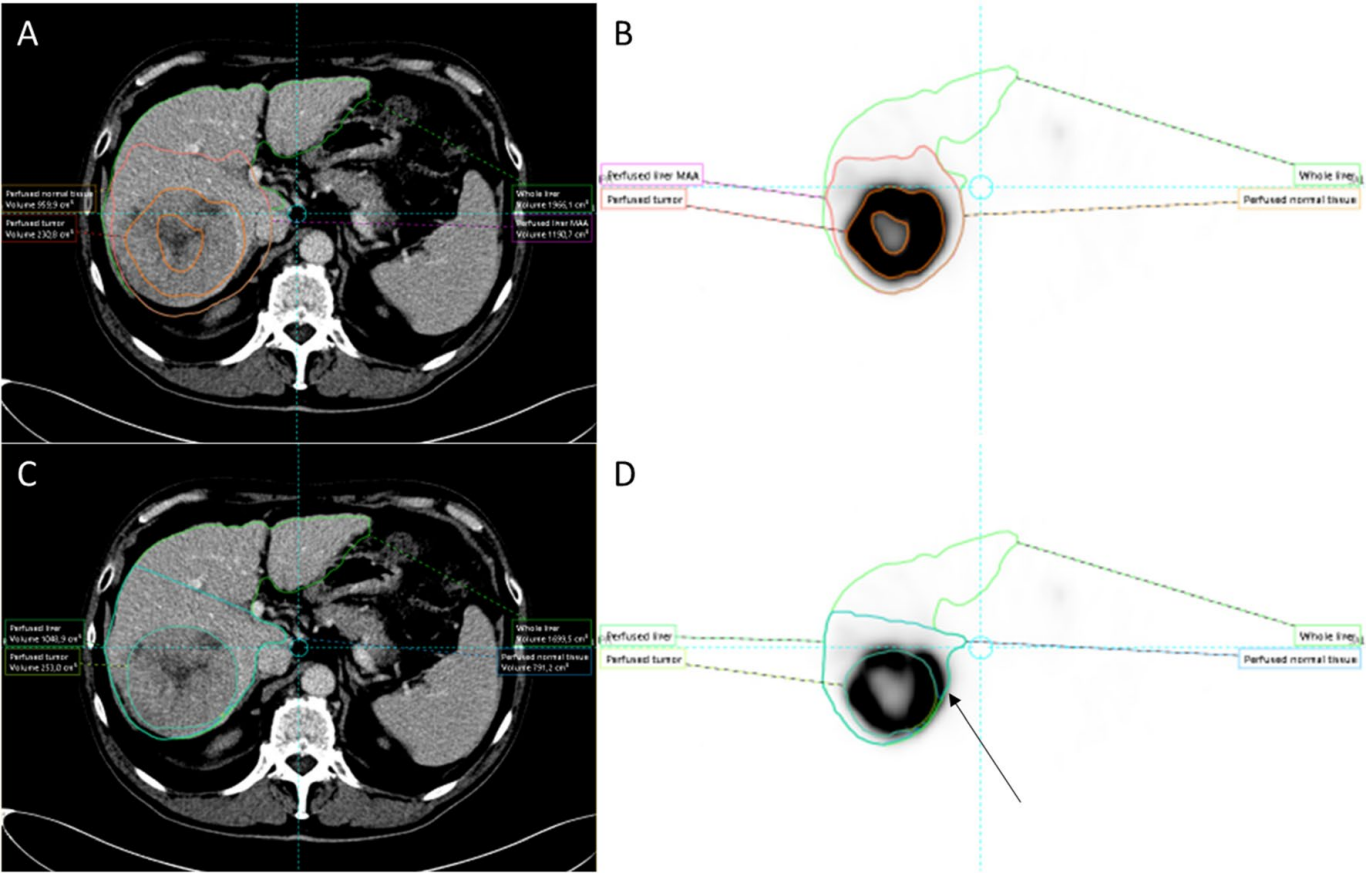
Well-defined high ^99m^Tc-MAA accumulation in and around the tumour favors threshold-based segmentation on SPECT (panels **A** and **B**) over CT-based segmentation (panels **C** and **D**) because threshold-based segmentation automatically excludes central necrosis in this case and overcomes misalignment issues between CT and SPECT (arrow). Note: perfused volume definition between both methods is similar

**Fig. 4:**
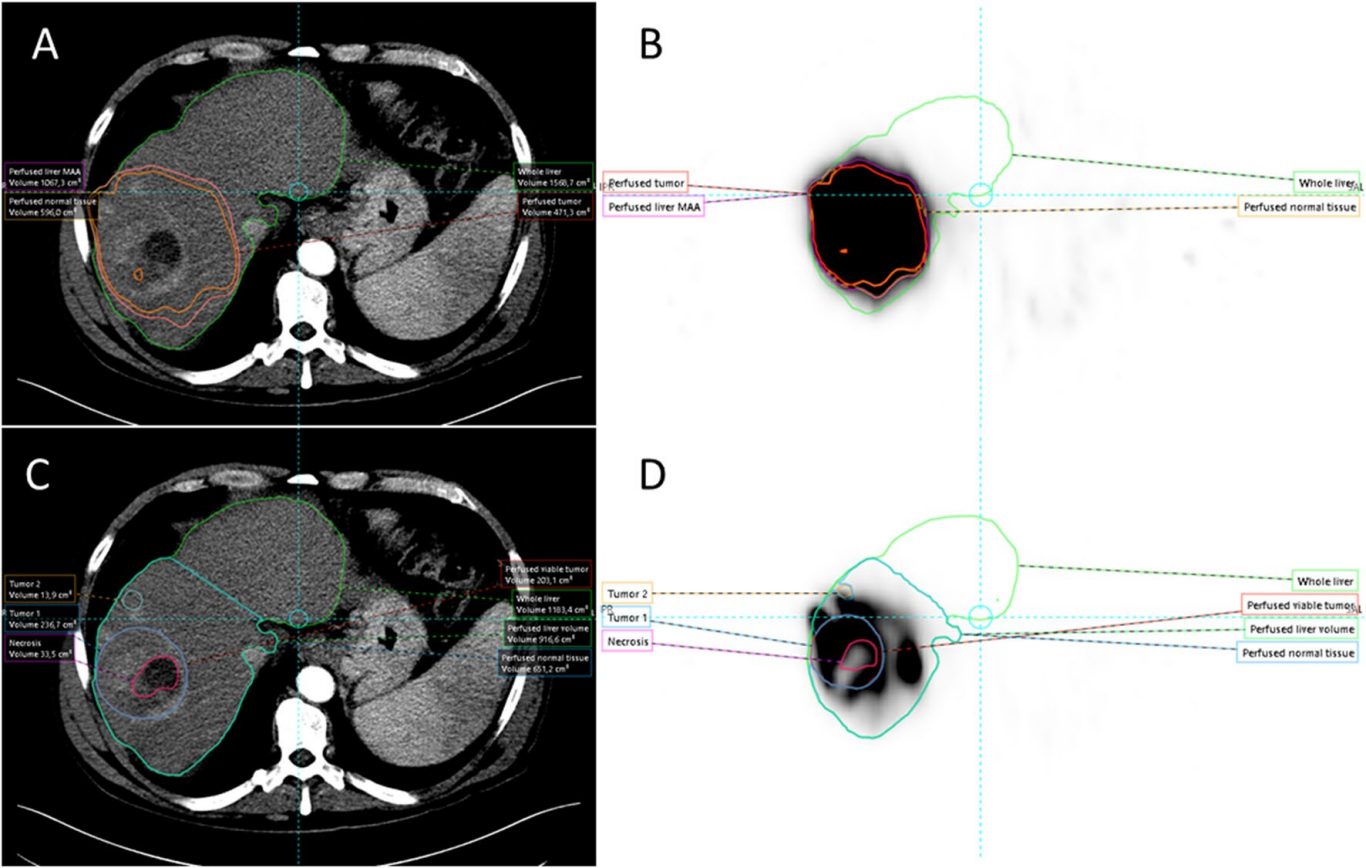
Heterogeneous ^99m^Tc-MAA in and around the tumour limits threshold-based segmentation on SPECT (panels **A** and **B**), where CT-based segmentation (panels **C** and **D**) better captures the contrast-enhancing tumour, central necrosis, and satellite lesions. Note: a difference in perfused volume definition between both methods is also present

**Fig. 5:**
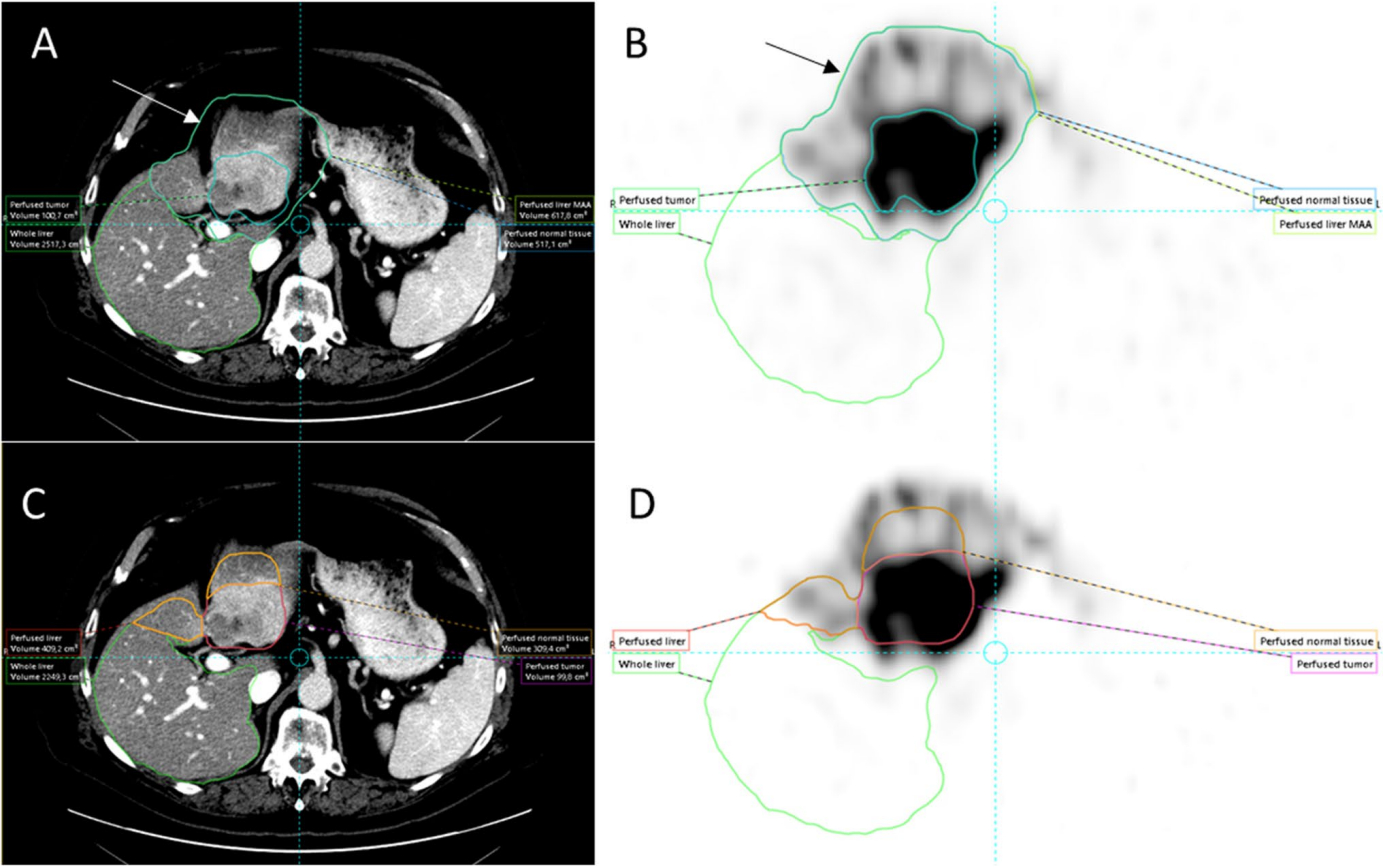
Threshold-based segmentation of the tumour on SPECT (panels **A** and **B**) is similar to CT-based segmentation (panels **C** and **D**) because of well-defined.^99m^Tc-MAA accumulation in the tumour and contrast enhancement on CT. Both methods work in this case. Note: perfused volume definition between both methods is very different with overestimation on SPECT (arrows)

For all dosimetric endpoints, inter-observer reproducibility was better for anatomic segmentation (RDC range: 1.17 to 3.53) than for ^99m^Tc-MAA SPECT segmentation (RDC range: 1.29 to 7.00) and similar for CT (RDC range: 1.09 to 3.56) and MRI (RDC range: 1.24 to 3.50); see Table 1 for specific RDC values and 95% CIs. Moreover, inter-observer reproducibility was better for larger volumes. Whole liver volume RDCs were 1.17 and 1.29 for anatomic and ^99m^Tc-MAA SPECT segmentation, with corresponding RDCs for whole liver absorbed doses of 1.25 and 1.97, respectively. Perfused normal tissue volume RDC was 1.95 for anatomic and 3.19 for ^99m^Tc-MAA SPECT segmentation, with corresponding RDCs for absorbed doses of 1.46 and 1.75. The effect of smaller volumes on the magnitude of RDC was emphasized by the total perfused tumour volume, where RDC was 2.92 for anatomic and 7.00 for ^99m^Tc-MAA SPECT with corresponding absorbed dose RDC of 1.84 and 2.78. These differences in inter-observer reproducibility between the different dosimetric endpoints are more easily seen when categorized into different “grades of reproducibility,” based on the upper value of the 95% CI for the RDC (Table 2) and as bar charts of mean and standard deviation over the reviewers separately for each of the 20 patients (Fig. 2). Normal tissue volume and absorbed dose calculations, usually encompassing a large(r) volume, showed low inter-observer variability. Medians over the 8 reviewers for perfused normal tissue volume ranged between 430.8 and 1548.8 cm^3^ for anatomic segmentation and between 331.9 and 1660.1 cm^3^ for ^99m^Tc-MAA SPECT segmentation for the 20 patients. Smaller tumourous structures showed considerable variation between reviewers, with medians for total perfused tumour volume ranging between 29.5 and 1181.2 cm^3^ for anatomic segmentation and ranging between 19.3 and 539.2 cm^3^ for ^99m^Tc-MAA SPECT segmentation. MAPE and CV% followed similar trends (provided in Supplementary tables). Figures 3, 4, and 5 illustrate the segmentation methods on imaging in case examples.

**Table 2:** Reproducibility coefficient (RDC*) grades for reproducibility

Grade	Upper value of 95% CI for RDC*	Segmentation	Administered activity	Whole liver volume	Whole liver absorbed dose	Whole liver normal tissue volume	Whole liver NTAD	Perfused normal tissue volume	Perfused NTAD	Total perfused tumour volume	Total perfused TAD
1	< 1.5	Anatomic	X	X	X						
		^99m^Tc-MAA SPECT									
2	≥ 1.5 and < 2	Anatomic				X					
		^99m^Tc-MAA SPECT		X		X					
3	≥ 2 and < 3	Anatomic					X	X	X		X
		^99m^Tc-MAA SPECT			X				X		
4	≥ 3	Anatomic								X	
		^99m^Tc-MAA SPECT	X				X	X		X	X

*CI*, confidence interval; *RDC*, reproducibility coefficient; ^*99m*^*Tc-MAA*, technetium-99m macroaggregated albumin; *NTAD*, normal tissue absorbed dose; *SPECT*, single-photon emission computed tomography; *TAD*, tumour absorbed dose

^*^Corresponding to the maximum fold difference in the measurement from two different reviewers in 95% of cases

Overall results for intra-observer reproducibility were consistent with inter-observer reproducibility. For most of the dosimetric endpoints, intra-observer reproducibility was better for anatomic segmentation than for ^99m^Tc-MAA SPECT segmentation. Also, intra-observer variability (IOV) was better for larger volumes. Whole liver volume IOV was 107.8 cm^3^ and 142.5 cm^3^ for anatomic and ^99m^Tc-MAA SPECT segmentation, with corresponding whole liver absorbed dose IOV of 8 Gy and 10.7 Gy, respectively. Perfused normal tissue volume IOV was 268.6 cm^3^ for anatomic and 231.4 cm^3^ for ^99m^Tc-MAA SPECT segmentation, with corresponding IOV for absorbed doses of 16.7 Gy and 19.7 Gy. In contrast, total perfused tumour volume IOV was 226.2 cm^3^ for anatomic and 153.3 cm^3^ for ^99m^Tc-MAA SPECT with corresponding absorbed dose IOV of 50.8 Gy and 121.4 Gy (Table 3). Interestingly, while IOV was (unexpectedly, and in contrast with inter-observer reproducibility) higher for anatomic segmentation in some volume calculations (e.g., perfused normal tissue volume, total perfused tumour volume), this did not translate into higher IOV for absorbed dose calculations, which were better for anatomic segmentation.

**Table 3:** Intra-observer variability (IOV) (see Supplementary information for definition)

	^99m^Tc-MAA SPECT segmentation, *n* = 160^a^	Anatomic segmentation, *n* = 160^a^
Administered activity (GBq)	0.6	0.3
Whole liver volume (cm^3^)	142.5	107.8
Whole liver absorbed dose (Gy)	10.7	8.0
Perfused liver volume (cm^3^)	236.1	133.5
Total perfused tumours volume (cm^3^)	153.3	226.2
Total perfused tumours absorbed dose (Gy)	121.4	50.8
Perfused normal tissue volume (cm^3^)	231.4	268.6
Perfused normal tissue absorbed dose (Gy)	19.7	16.7
Target lesion volume (cm^3^)	146.2	226.8
Target lesion absorbed dose (Gy)	122.3	58.8

^*99m*^*Tc-MAA*, technetium-99 macroaggregated albumin; *SPECT*, single-photon emission computed tomography

^a^*n* = 160 (i.e., 10 patients × 8 reviewers × 2 assessments); *n* = 136 for target lesion volume and target lesion absorbed dose

Outliers were evenly distributed between anatomic (*n* = 24) and ^99m^Tc-MAA segmentation (*n* = 24). For ^99m^Tc-MAA segmentation, 23/24 outliers were from a single reviewer. This was related to a higher set threshold value. Outliers for anatomic segmentation were noted for 6/8 reviewers, the majority from two reviewers (*n* = 14) with no particular identified pattern. Higher numbers of outliers were noted (*n* = 36) for first assessments than for second assessments (*n* = 12), although second assessments were performed for half of the patients (*n* = 10) and may be related to familiarity with individual patient imaging from the first assessment. More outliers were identified in assessments of total perfused TAD (*n* = 8) or total perfused tumour volume (*n* = 5) than for total perfused NTAD (*n* = 2) or total perfused normal tissue volume (*n* = 4).

## Discussion

Numerous publications evaluated a variety of methods focused on reproducibility of ^99m^Tc-MAA as a surrogate for TAD and NTAD, compared pre-treatment dosimetry using ^99m^Tc-MAA and post-treatment dosimetry of ^90^Y, and evaluated safety and efficacy outcomes based on pre-treatment ^99m^Tc-MAA and post-treatment ^90^Y dosimetry [4, 7, 9–11, 15–17, 19, 21, 22]. While a variety of reproducibility measures were evaluated, consensus was noted in better reproducibility of NTAD versus TAD [4, 7, 10, 16, 17, 19, 21, 22]. Despite the lower reproducibility of TAD, estimation using ^99m^Tc-MAA and/or post ^90^Y PET proved to be reliable measures to predict efficacy outcomes [4, 7, 9, 11, 15, 16, 19, 21]. A comparison of the two used segmentation methods based on either ^99m^Tc-MAA or anatomic CT/MRI was not previously reported and was the basis of the current study. In summary, ^99m^Tc-MAA-based segmentation resulted in higher values for both TAD and NTAD, but inferior inter- and intra-observer reproducibility. The retrospective dosimetry analysis assumed 120 Gy to the perfused volume for all patients (i.e., similar administered activity). As the ^99m^Tc-MAA segmentation method identified smaller tumours and normal tissue perfused volumes, the TAD and NTAD were higher. Independent of the segmentation technique, larger volumes lead to superior reproducibility compared with smaller volumes.

In the TARGET clinical evaluation substudy, anatomic segmentation was used, while in the DOSISPHERE-01 study, the ^99m^Tc-MAA-based segmentation method was used [7, 9]. Although the anatomic segmentation method exhibited better reproducibility, the findings of DOSISPHERE-01 (and other studies) do support the clinical utility of ^99m^Tc-MAA-based multicompartment dosimetry for treatment planning [9–11, 18, 19, 21]. It may be hypothesized that the acceptability of ^99m^Tc-MAA-based segmentation, at least in part, relies on the size and hypervascularity of the treated lesions (as in the DOSISPHERE-01 study), which may increase reproducibility and the predictive value of ^99m^Tc-MAA. In fact, reproducibility was better for both segmentation methods for larger volumes compared with smaller volumes, most notably larger normal tissue volumes versus (usually) smaller tumour volumes [7, 9, 10, 15]. Although greater variability was noted for tumour volume and absorbed dose, both the anatomic and ^99m^Tc-MAA SPECT segmentation methods have demonstrated statistical association of TAD with tumour response and increased OS and provide a clinically reliable estimation of ^90^Y glass microsphere treatment outcomes [7, 9, 15].

The anatomic segmentation method, previously reported for the retrospective TARGET clinical evaluation substudy, may be more easily adopted as it defines volume on diagnostic imaging and is the backbone of tumour response assessment [7]. Anatomic segmentation preference is supported by the lower variability found in this study for treatment planning with ^90^Y glass microspheres. It may also be preferred when the catheter location for treatment is adjusted based on ^99m^Tc-MAA distribution. This may lead to changes in perfused volumes and perfused total tumour volumes that do not necessarily correlate with the ^99m^Tc-MAA distribution.

^99m^Tc-MAA-based SPECT segmentation uses a ^99m^Tc-MAA count-based threshold, where higher focal counts are associated with tumours and lower counts with normal tissue. This count-based threshold was selected by individual reviewers, such that total ^99m^Tc-MAA counts were confined within the perfused volume and highest counts within the tumours, under the assumption that all high count areas involved tumours (which may not always be the case) [9, 18]. In the majority of cases, the anatomic method may, therefore, be best suited for assessment; however, in selected cases, ^99m^Tc-MAA SPECT segmentation may be the preferred basis for assessment, with consultation of anatomic images for reference. These selected cases may include (1) cases with uncertain or poor quality of registration (for which anatomic delineated volumes of interest will not accurately capture all the ^99m^Tc-MAA counts in these volumes), (2) cases with significant areas of tumour necrosis (that are not always easily defined on anatomic imaging but lack ^99m^Tc-MAA counts and are therefore easily segmented on ^99m^Tc-MAA SPECT), and (3) cases with a significant discrepancy between perfused volumes defined according to anatomy and ^99m^Tc-MAA count distribution (for which ^99m^Tc-MAA distribution more accurately represents the actual perfused volume). Segmentation method choice should be based on disease presentation, image availability and quality, and reviewer familiarity and preference. Although the association between TAD and tumour response and OS, and NTAD and toxicity, holds for both anatomic and ^99m^Tc-MAA SPECT segmentation, the used dose thresholds will be different. Dose–effect relationships, therefore, depend on clinical parameters on the one hand (e.g., tumour type, clinical setting), but on technical parameters on the other hand (e.g., a segmentation method, acquisition parameters, pre- versus post-treatment imaging).

Reproducibility, in general, and by individual practitioners will improve with familiarity and utilization of the information obtained with both segmentation methods. The authors recommend assessing both segmentation methods in a hybrid approach to best identify the appropriate dosimetry in individual patients. Recommended dose thresholds should be used according to the clinical and technical parameters provided or should be adjusted to the case at hand. Future research should focus on refining dose threshold recommendations according to these clinical and technical parameters.

Similar to prior studies summarized in Table 4, inter-observer investigation demonstrated better reproducibility for larger volumes, i.e., whole liver and normal tissue [19–21]. This finding is consistent with factors contributing to increased variability, primarily driven by the partial volume effect [18, 20]. Despite the similarities in RDC values for whole liver volumes, the variability was higher for whole liver and whole liver NTAD for ^99m^Tc-MAA SPECT segmentation versus anatomic segmentation. Perfused normal tissue volume and NTAD also demonstrated better reproducibility for anatomic segmentation versus ^99m^Tc-MAA SPECT segmentation. Nonetheless, better reproducibility for NTAD is noted by both segmentation methods versus TAD and confirms single-center assessments of multicompartment dosimetry for NTAD being the appropriate choice as the key safety factor [7, 9–11, 16–19, 21].

**Table 4:** Reproducibility coefficient (RDC) values and grade from TARGET round robin substudy compared with published data

Study/publication	Number of patients; number of reviewers	Imaging modality	RDC (95% CI) for NTAD	RDC (95% CI) for TAD
Haste et al., .2017 [22]	73 and 63 patients^a^ 3 reviewers	^99m^Tc-MAA SPECT ^b^	WLNT (grade 2)^d^ 1.4 (1.4 to 1.5)	Tumour (grade 4)^d^ 2.4 (2.2 to 3.2)
		^90^Y PET	WLNT (grade 2)^d^ 1.4 (1.3 to 1.6)	Tumour (grade 2)^d^ 1.6 (1.5 to 1.8)
Meyers et al., 2020 [19]	23 patients 3 reviewers	^90^Y PET	WLNT (grade 2)^d^ 1.33 (1.26 to 1.68)	Tumour (grade 3)^d^ 1.52 (1.38 to 2.25)
TARGET round robin	20 patients 8 reviewers	^99m^Tc-MAA SPECT segmentation ^c^	WLNT (grade 4)^d^ 3.62 (2.98 to 6.95) Perfused normal tissue (grade 3)^d^ 1.75 (1.62 to 2.31)	Total perfused tumours (grade 4)^d^ 2.78 (2.29 to 5.24) Target lesion (grade 4)^d^ 2.84 (2.33 to 5.41)
		Anatomic segmentation ^c^	WLNT (grade 3)^d^ 1.85 (1.69 to 2.52) Perfused normal tissue (grade 3)^d^ 1.46 (1.39 to 2.25)	Total perfused tumours (grade 3)^d^ 1.84 (1.66 to 2.56) Target lesion (grade 4)^d^ 2.07 (1.87 to 3.10)

*WLNT*, whole liver normal tissue

^a^WLNT absorbed dose was assessed in 73 patients; TAD was assessed in 63 patients, with only the largest tumour being assessed for patients with multiple tumours

^b^Haste et al. used ^99m^Tc-MAA SPECT rather than ^99m^Tc-MAA SPECT/CT

^c^TARGET round robin used ^99m^Tc-MAA SPECT/CT and diagnostic CT or MRI imaging

^d^Grade assigned based on Table 2

Contributions to increased variability are likely related to tumour characteristics (e.g., size, vascularity, necrosis, PVT thrombus, infiltration), image quality, partial volume effect, variability in ^99m^Tc-MAA distribution, registration error, and differences in perfused versus anatomic volumes measured via anatomic or ^99m^Tc-MAA SPECT segmentation [17, 19–22]. Most factors seem to limit the ^99m^Tc-MAA SPECT segmentation method more than the anatomic method and seem to impact smaller volumes more than larger volumes. One exception is the registration error between ^99m^Tc-MAA SPECT and CT/MRI, which may lead to increased variability using the anatomic segmentation method (Fig. 3), especially in the case of multiple smaller tumours. In individual cases where misregistration is clearly present, the ^99m^Tc-MAA SPECT segmentation method may be preferred. In the current study, however, this effect did not outweigh the other effects on the variability that consistently favored the anatomic method.

DOSISPHERE-01, a randomized controlled study, prospectively demonstrated that ^99m^Tc-MAA SPECT segmentation for multicompartment dosimetry could be successfully applied in HCC patients, resulting in improved tumour response and OS. In DOSISPHERE-01, patient inclusion, selection, and planning were based on the ^99m^Tc-MAA SPECT segmentation method. Patients, however, had large tumours (mean index tumour size of 10.6 cm and 11.1 cm in the two arms of the study), which is usually sufficient for a well-developed vascular supply that typically results in a higher tumour to normal tissue ^99m^Tc-MAA distribution ratio, ideal for the ^99m^Tc-MAA SPECT segmentation method. The round robin substudy and associated TARGET study included a significantly different population, also including smaller and less hypervascular tumours [20]. These real-world data highlight the differences in individual patients and the subsequent need to evaluate both anatomic and ^99m^Tc-MAA SPECT segmentation methods for optimal treatment planning. Reproducibility is expected to improve for anatomic and ^99m^Tc-MAA SPECT segmentation as physicians implement proper angiography techniques (e.g., catheter positioning, C-arm CT), gain experience with dosimetry software and multicompartment dosimetry, and identify how best to utilize the two segmentation methods individually or in a hybrid approach, using aspects of both segmentation methods to further improve patient outcomes [7, 9, 17, 19–21].

Limitations of this study include reviewers’ enhanced familiarity with the anatomic method, which may have contributed to the higher variability in ^99m^Tc-MAA SPECT segmentation. Following prespecified statistical analysis, individual data points were statistically identified for both inter- and intra-observer results as outliers; the bulk of which were from a single site and related to a difference in segmentation instruction interpretation specific to the ^99m^Tc-MAA SPECT segmentation. A learning curve may also have been attributed to the variability of outlier occurrence and inter- and intra-observer differences. Furthermore, the limited sample size did not allow for the analysis of factors contributing to higher variability. Intra-observer variability was studied as an exploratory analysis on 10 patients only and may consequently have been underpowered. Nevertheless, the results were consistent throughout.

Although the anatomic method performed better than the ^99m^Tc-MAA SPECT segmentation method, the anatomic method may not be universally preferred. Future research should focus on optimal methods for each patient case. To appreciate published dose–effect relationships and reported dose thresholds and implement these results in clinical guidelines, it is important to have a clear understanding of the method used. In applying the anatomic or ^99m^Tc-MAA SPECT segmentation method, differences between the estimated TAD and NTAD should be taken into account. For standardization purposes, when introducing multiple dosimetric methods in the same population, one must establish a comprehensive decision algorithm to decide which should be applied in which scenario.

## Conclusion

Compared with ^99m^Tc-MAA segmentation, anatomic segmentation-based dosimetry results in lower absorbed doses with superior inter- and intra-observer reproducibility. Higher (normal liver) volume compartments yield the most reproducible results. Most likely, the preferred method should be decided on a case-by-case basis.

### Supplementary Information

Below is the link to the electronic supplementary material.

Supplementary file1 (PDF 272 KB)

## Abbreviations

**NTAD**: Normal tissue absorbed dose
**TAD**: Tumour absorbed dose
**HCC**: Hepatocellular carcinoma
^**90**^**Y**: Yttrium-90
**TARGET**: The TheraSphere™ Advanced Dosimetry Retrospective Global Study Evaluation in Hepatocellular Carcinoma Treatment
**AE**: Adverse event
^**99m**^**Tc****-MAA**: Technetium-99 macroaggregated albumin
**RDC**: Reproducibility coefficient
**LoA**: Limits of agreement
**SPECT/CT**: Single-photon emission computed tomography/computed tomography
**MRI**: Magnetic resonance imaging
**TARE**: Transarterial radioembolization
**OS**: Overall survival
**MIRD**: Medical internal radiation dose
**PET/CT**: Positron emission tomography/computed tomography
**PVT**: Portal vein thrombosis
**CV**: Coefficient of variation
**CI**: Confidence interval
**MAPE**: Mean absolute percentage error
**IOV**: Intra-observer variability
**WLNT**: Whole liver normal tissue

## References

[1] Salem R, Padia SA, Lam M, Bell J, Chiesa C, Fowers K (2019). Clinical and dosimetric considerations for Y90: recommendations from an international multidisciplinary working group. Eur J Nucl Med Mol Imaging.

[2] d'Abadie P, Walrand S, Hesse M, Annet L, Borbath I, Van den Eynde M (2021). Prediction of tumor response and patient outcome after radioembolization of hepatocellular carcinoma using 90Y-PET-computed tomography dosimetry. Nucl Med Commun.

[3] Chan KT, Alessio AM, Johnson GE, Vaidya S, Kwan SW, Monsky W (2018). Prospective trial using internal pair-production positron emission tomography to establish the yttrium-90 radioembolization dose required for response of hepatocellular carcinoma. Int J Radiat Oncol Biol Phys.

[4] Kappadath SC, Mikell J, Balagopal A, Baladandayuthapani V, Kaseb A, Mahvash A (2018). Hepatocellular carcinoma tumor dose response after (90)Y-radioembolization with glass microspheres using (90)Y-SPECT/CT-based voxel dosimetry. Int J Radiat Oncol Biol Phys.

[5] Gabr A, Kulik L, Mouli S, Riaz A, Ali R, Desai K (2021). Liver transplantation following yttrium-90 radioembolization: 15-year experience in 207-patient cohort. Hepatology.

[6] Garin E, Rolland Y, Pracht M, Le Sourd S, Laffont S, Mesbah H (2017). High impact of macroaggregated albumin-based tumour dose on response and overall survival in hepatocellular carcinoma patients treated with (90) Y-loaded glass microsphere radioembolization. Liver Int.

[7] Lam M, Garin E, Maccauro M, Kappadath SC, Sze DY, Turkmen C (2022). A global evaluation of advanced dosimetry in transarterial radioembolization of hepatocellular carcinoma with Yttrium-90: the TARGET study. Eur J Nucl Med Mol Imaging.

[8] Matsumoto MM, Mouli S, Saxena P, Gabr A, Riaz A, Kulik L (2021). Comparing real world, personalized, multidisciplinary tumor board recommendations with BCLC Algorithm: 321-patient analysis. Cardiovasc Intervent Radiol.

[9] Garin E, Tselikas L, Guiu B, Chalaye J, Edeline J, de Baere T (2021). Personalised versus standard dosimetry approach of selective internal radiation therapy in patients with locally advanced hepatocellular carcinoma (DOSISPHERE-01): a randomised, multicentre, open-label phase 2 trial. Lancet Gastroenterol Hepatol.

[10] Chiesa C, Mira M, Bhoori S, Bormolini G, Maccauro M, Spreafico C (2020). Radioembolization of hepatocarcinoma with (90)Y glass microspheres: treatment optimization using the dose-toxicity relationship. Eur J Nucl Med Mol Imaging.

[11] d'Abadie P, Walrand S, Lhommel R, Hesse M, Jamar F. A theranostic approach in SIRT: value of pre-therapy imaging in treatment planning. J Clin Med. 2022;11:7245. 10.3390/jcm11237245. 10.3390/jcm11237245 PMC9736029 36498819

[12] Chiesa C, Mira M, Maccauro M, Romito R, Spreafico C, Sposito C (2012). A dosimetric treatment planning strategy in radioembolization of hepatocarcinoma with 90Y glass microspheres. Q J Nucl Med Mol Imaging.

[13] Garin E, Lenoir L, Edeline J, Laffont S, Mesbah H, Poree P (2013). Boosted selective internal radiation therapy with 90Y-loaded glass microspheres (B-SIRT) for hepatocellular carcinoma patients: a new personalized promising concept. Eur J Nucl Med Mol Imaging.

[14] Garin E, Lenoir L, Rolland Y, Edeline J, Mesbah H, Laffont S (2012). Dosimetry based on 99mTc-macroaggregated albumin SPECT/CT accurately predicts tumor response and survival in hepatocellular carcinoma patients treated with 90Y-loaded glass microspheres: preliminary results. J Nucl Med.

[15] Ho CL, Chen S, Cheung SK, Leung YL, Cheng KC, Wong KN (2018). Radioembolization with (90)Y glass microspheres for hepatocellular carcinoma: significance of pretreatment (11)C-acetate and (18)F-FDG PET/CT and posttreatment (90)Y PET/CT in individualized dose prescription. Eur J Nucl Med Mol Imaging.

[16] Jadoul A, Bernard C, Lovinfosse P, Gérard L, Lilet H, Cornet O (2020). Comparative dosimetry between (99m)Tc-MAA SPECT/CT and (90)Y PET/CT in primary and metastatic liver tumors. Eur J Nucl Med Mol Imaging.

[17] Kafrouni M, Allimant C, Fourcade M, Vauclin S, Guiu B, Mariano-Goulart D (2019). Analysis of differences between (99m)Tc-MAA SPECT- and (90)Y-microsphere PET-based dosimetry for hepatocellular carcinoma selective internal radiation therapy. EJNMMI Res.

[18] Chiesa C, Sjogreen-Gleisner K, Walrand S, Strigari L, Flux G, Gear J (2021). EANM dosimetry committee series on standard operational procedures: a unified methodology for 99mTc-MAA pre- and 90Y peri-therapy dosimetry in liver radioembolization with 90Y microspheres. EJNMMI Phys.

[19] Meyers N, Jadoul A, Bernard C, Delwaide J, Lamproye A, Detry O (2020). Inter-observer variability of (90)Y PET/CT dosimetry in hepatocellular carcinoma after glass microspheres transarterial radioembolization. EJNMMI Phys.

[20] Covert EC, Fitzpatrick K, Mikell J, Kaza RK, Millet JD, Barkmeier D (2022). Intra- and inter-operator variability in MRI-based manual segmentation of HCC lesions and its impact on dosimetry. EJNMMI Phys.

[21] Thomas MA, Mahvash A, Abdelsalam M, Kaseb AO, Kappadath SC (2020). Planning dosimetry for (90) Y radioembolization with glass microspheres: evaluating the fidelity of (99m) Tc-MAA and partition model predictions. Med Phys.

[22] Haste P, Tann M, Persohn S, LaRoche T, Aaron V, Mauxion T (2017). Correlation of technetium-99m macroaggregated albumin and yttrium-90 glass microsphere biodistribution in hepatocellular carcinoma: a retrospective review of pretreatment single photon emission CT and posttreatment positron emission tomography/CT. J Vasc Interv Radiol.

[23] Raunig DL, McShane LM, Pennello G, Gatsonis C, Carson PL, Voyvodic JT (2015). Quantitative imaging biomarkers: a review of statistical methods for technical performance assessment. Stat Methods Med Res.

