# Supplementary material for: Direct comparison and reproducibility of two segmentation methods for multicompartment dosimetry: round robin study on radioembolization treatment planning in hepatocellular carcinoma
Source: Eur J Nucl Med Mol Imaging. 2023 Sep 12;51(1):245–57. doi: 10.1007/s00259-023-06416-9 (PMC10684706; doi:10.1007/s00259-023-06416-9)
Supplement: Supplementary file 1 — Supplementary file1 (PDF 272 KB) [file 259_2023_6416_MOESM1_ESM.pdf]

Supplementary Information

Round Robin study on radioembolization treatment planning in hepatocellular carcinoma: Direct comparison and reproducibility of multicompartment dosimetry.

Marnix Lam[1]; Etienne Garin[2]; Xavier Palard-Novello[2]; Armeen Mahvash[3]; Cheenu Kappadath[3]; Paul Haste[4]; Mark Tann[4]; Ken Herrmann[5]; Francesco Barbato[5]; Brian Geller[6]; Niklaus Schaefer[7]; Alban Denys[8]; Matthew Dreher[9]; Kirk D. Fowers[9]; Vanessa Gates[10]; Riad Salem[10]

1. Department of Radiology and Nuclear Medicine, University Medical Center Utrecht, The Netherlands
2. Nuclear Medicine Department, Eugene Marquis Center, Rennes, France
3. Department of Interventional Radiology, University of Texas MD Anderson Cancer Center, Houston, TX, USA
4. Department of Clinical Radiology and Imaging Sciences, Indiana University School of Medicine, Indianapolis, IN, USA
5. Department of Nuclear Medicine, University of Duisburg-Essen, and German Cancer Consortium (DKTK)-University Hospital Essen, Essen, Germany
6. Department of Radiology, University of Florida, Gainesville, FL, USA
7. Department of Nuclear Medicine and Molecular Imaging, Lausanne University Hospital CHUV, University of Lausanne, Lausanne, Switzerland
8. Department of Radiology and Interventional Radiology, Lausanne University Hospital CHUV, University of Lausanne, Lausanne, Switzerland
9. Boston Scientific Corporation, Marlborough, MA, USA
10. Department of Radiology, Northwestern Feinberg School of Medicine, Chicago, IL, USA

Corresponding Author: Marnix Lam (m.lam@umcutrecht.nl)

Table of Contents

Computation of Intra-observer Reproducibility

Table S1 Inter-Observer Coefficient of Variation (CV), % Values

Table S2 Inter-Observer Mean Absolute Percentage Error (MAPE) Values

Computation of Intra-observer Reproducibility

The intra-observer variability (IOV) is the measurement of the variation between readings of the same patient by the same observer.

The following statistical model was used. Let the dosimetric endpoint for the ith patient assessed by the sth reviewer the jth time be

$$y_{ijs} = \mu + \gamma_i + \delta_s + (\gamma\delta)_{is} + \eta_j + \epsilon_{ijs}$$

where

$\gamma_i \sim$ Normal (0, σ_γ^2) for patient i = 1, …, n (n = 10 = total number of patients)

$\delta_s \sim$ Normal (0, σ_δ^2) for reviewer s = 1, 2, 3,…, 8

η_j is a fixed effect for review sequence j = 1, 2 (review sequence 1st, 2nd)

$(\gamma\delta)_{is} \sim$ Normal (0, $\sigma_{\gamma\delta}^2$) is the interaction effect between patient i and reviewer s

$\epsilon_{ijs} \sim$ Normal (0, σ_ε^2).

The IOV was assessed by estimating (1a) the intra-observer variance and (1b) the intra-observer mean square error. The intra-observer variance was estimated by $\hat{\sigma}_\varepsilon^2$. The intra-observer mean square error was obtained by $(\hat{\eta}_2 - \hat{\eta}_1)^2 + 2\hat{\sigma}_\varepsilon^2$. The fixed effect $(\hat{\eta}_2 - \hat{\eta}_1)$ and the variance component $(\hat{\sigma}_\varepsilon^2)$ were obtained using the SAS Proc Mixed procedure. IOV was computed as the square root of the sum of (1a) and (1b).

SUPPLEMENT Table S1. Inter-Observer Coefficient of Variation (CV), % Values

Parameter	Segmentation Method				
		^{99m}MAA SPECT	Anatomic CT	Anatomic MRI	Anatomic CT/MRI
**Whole Liver**					
Volume (cm^3)	n	20	12	8	20
	Mean	8.47	2.70	7.86	4.76
	Median	8.14	2.12	7.33	3.88
	Min, Max	4.9, 18.4	0.7, 8.0	4.9, 11.4	0.7, 11.4
Absorbed Dose (Gy)	n	20	12	8	20
	Mean	19.78	8.09	6.52	7.46
	Median	18.75	7.61	5.38	6.57
	Min, Max	6.8, 41.6	4.4, 12.8	2.5, 14.4	2.5, 14.4
**Perfused Liver**[a]					
Volume (cm^3)	n	20	12	8	20
	Mean	21.47	8.82	8.70	8.77
	Median	22.07	9.43	8.19	9.43
	Min, Max	6.7, 42.8	4.7, 13.1	3.9, 15.0	3.9, 15.0
**Total Perfused Tumours**					

Volume (cm^3)	n	20	12	8	20
	Mean	55.94	40.35	49.71	44.09
	Median	50.77	40.10	49.12	46.16
	Min, Max	26.6, 180.9	30.5, 49.7	30.1, 71.8	30.1, 71.8
Absorbed Dose (Gy)	n	20	12	8	20
	Mean	38.54	16.83	23.40	19.46
	Median	35.03	15.53	23.55	18.02
	Min, Max	12.1, 105.5	7.9, 30.4	8.5, 38.0	7.9, 38.0
**Perfused Normal Tissue**					
Volume (cm^3)	n	20	12	8	20
	Mean	30.28	16.58	18.64	17.41
	Median	30.33	14.00	14.39	14.00
	Min, Max	9.4, 54.4	5.5, 42.2	7.2, 40.1	5.5, 42.2
Absorbed Dose (Gy)	n	20	12	8	20
	Mean	17.29	12.68	9.63	11.46
	Median	15.49	12.70	6.42	10.25
	Min, Max	3.6, 42.8	0.6, 22.9	0.7, 28.7	0.6, 28.7
**Whole Liver**					

**Normal Tissue**					
Volume (cm^3)	n	20	12	8	20
	Mean	10.37	6.99	11.83	8.93
	Median	9.82	3.57	11.86	7.17
	Min, Max	4.8, 18.8	1.8, 30.6	6.1, 18.0	1.8, 30.6
Absorbed Dose (Gy)	n	20	12	8	20
	Mean	35.44	19.51	16.85	18.45
	Median	35.98	18.51	13.73	17.21
	Min, Max	10.2, 68.9	4.3, 44.1	2.8, 34.3	2.8, 44.1
**Target Lesion**[b]					
Volume (cm^3)	n	20	12	8	20
	Mean	55.36	33.52	40.77	36.42
	Median	52.15	23.70	40.39	27.08
	Min, Max	25.0, 173.8	9.3, 123.3	19.2, 77.6	9.3, 123.3
Absorbed Dose (Gy)	n	20	12	8	20
	Mean	37.53	17.73	29.25	22.34
	Median	37.49	15.33	27.62	20.42
	Min, Max	8.8, 104.4	7.5, 31.8	8.3, 60.0	7.5, 60.0

[a] Absorbed dose to the perfused liver was set to 120 Gy, and so this endpoint was not included in the table.

[b] Target lesion data for some sites and patients have been excluded because lesions may not have been identifieddduring data entry in the eCRF according to instructions provided by the sponsor.

Abbreviations: max = maximum; min = minimum; n = number of patients

Notes: Summary statistics are computed over all patients.

SUPPLEMENT Table S2. Inter-Observer Mean Absolute Percentage Error (MAPE) Values

Parameter	Segmentation Method				
		^{99m}MAA SPECT	Anatomic CT	Anatomic MRI	Anatomic CT/MRI
**Whole Liver**					
Volume (cm^3)	n	20	12	8	20
	Mean	6.47	2.05	5.98	3.62
	Median	5.96	1.67	6.51	2.92
	Min, Max	3.9, 14.4	0.5, 6.4	3.4, 8.3	0.5, 8.3
Absorbed Dose (Gy)	n	20	12	8	20
	Mean	15.25	6.40	4.82	5.77
	Median	14.38	5.80	4.35	5.19
	Min, Max	4.7, 36.7	3.7, 10.6	1.8, 10.9	1.8, 10.9
**Perfused Liver**[a]					
Volume (cm^3)	n	20	12	8	20
	Mean	16.73	6.90	6.21	6.63
	Median	16.79	7.13	5.95	6.90
	Min, Max	4.4, 38.2	3.3, 10.0	2.9, 11.5	2.9, 11.5
**Total Perfused Tumours**					
Volume (cm^3)	n	20	12	8	20

	Mean	41.18	17.79	30.26	22.78
	Median	38.23	15.70	27.30	18.92
	Min, Max	20.4, 111.2	5.1, 45.1	14.6, 54.2	5.1, 54.2
Absorbed Dose (Gy)	n	20	12	8	20
	Mean	26.73	13.11	17.86	15.01
	Median	27.73	11.49	16.38	14.44
	Min, Max	9.5, 64.2	6.4, 25.4	6.7, 32.3	6.4, 32.3
**Perfused Normal Tissue**					
Volume (cm^3)	n	20	12	8	20
	Mean	24.33	12.73	13.49	13.03
	Median	23.21	10.39	9.94	10.39
	Min, Max	7.2, 47.4	4.3, 32.1	5.5, 28.4	4.3, 32.1
Absorbed Dose (Gy)	n	20	12	8	20
	Mean	13.22	10.43	7.26	9.16
	Median	12.35	10.34	5.11	8.04
	Min, Max	3.2, 33.0	0.5, 19.4	0.5, 22.5	0.5, 22.5
**Whole Liver Normal Tissue**					
Volume (cm^3)	n	20	12	8	20
	Mean	7.98	5.47	8.59	6.72

	Median	7.63	3.14	8.59	5.16
	Min, Max	4.1, 14.8	1.2, 26.2	4.7, 12.5	1.2, 26.2
Absorbed Dose (Gy)	n	20	12	8	20
	Mean	27.55	15.09	12.53	14.06
	Median	28.03	13.72	9.57	12.87
	Min, Max	6.7, 57.1	3.9, 35.1	2.0, 24.6	2.0, 35.1
**Target Lesion**[b]					
Volume (cm^3)	n	20	12	8	20
	Mean	41.24	25.88	29.66	27.39
	Median	40.62	16.20	30.65	18.61
	Min, Max	20.2, 111.9	6.7, 103.1	14.4, 47.2	6.7, 103.1
Absorbed Dose (Gy)	n	20	12	8	20
	Mean	26.38	13.92	22.85	17.49
	Median	27.82	12.87	19.82	15.39
	Min, Max	7.0, 63.5	5.3, 25.1	6.3, 51.1	5.3, 51.1

a. Absorbed dose to the perfused liver was set to 120 Gy, and so this endpoint was not included in the table.

b. Target lesion data for some sites and patients have been excluded because lesions may not have been identifiedduring data entry in the eCRF according to instructions provided by the sponsor.

Abbreviations: max = maximum; min = minimum; n = number of patients

Notes: Summary statistics are computed over all patients.
